# Cellular and Molecular Changes Induced by Various Preservation Temperatures and Methods of Preservation in Renal Grafts and Other Solid Organ Grafts

**DOI:** 10.3390/ijms27031294

**Published:** 2026-01-28

**Authors:** Talal Shamma, Cora England, Tamara S. Ortas, Hasan Ali, George J. Dugbartey, Alp Sener

**Affiliations:** 1 Department of Microbiology & Immunology, Western University, London, ON N6A 5C1, Canada; 2 Matthew Mailing Center for Translational Transplant Studies, Western University, London Health Sciences Center, London, ON N6A 5A5, Canada; 3 Department of Transplant Research and Innovation, King Faisal Specialist Hospital and Research Centre, Riyadh 11211, Saudi Arabia; 4 Weill Cornell Medicine, Doha P.O. Box 24144, Qatar; 5 Department of Surgery, Western University, London, ON N6A 5A5, Canada; 6 Department of Physiology & Pharmacology, Accra College of Medicine, Accra P.O. Box CT9828, Ghana

**Keywords:** static cold storage (SCS), hypothermic oxygenated machine perfusion (HOPE), normothermic machine perfusion (NMP), subnormothermic machine perfusion (SNMP), transplantation, ischemia-reperfusion injury (IRI), cold ischemic time (CIT)

## Abstract

Kidney transplantation remains the ultimate treatment option for patients with end-stage renal disease. However, the global shortage in donor kidneys, exacerbated by challenges such as ischemia–reperfusion injury (IRI), reduces renal graft viability and negatively impacts post-transplant outcomes. Static cold storage, the gold standard of organ preservation, reduces metabolic demand but increases the risk of cold-induced mitochondrial dysfunction and IRI, especially in marginal kidneys. The introduction of machine perfusion techniques allows renal grafts and other solid organ grafts to be preserved at a wider range of temperatures. Organ preservation temperatures play an important role in determining post-transplant outcomes in the transplantation of the kidney and other transplantable solid organs. Therefore, determining the optimal preservation temperature may help increase organ utilization by avoiding unnecessary graft discards and increasing the safe use of marginal organs. This review discusses the impact of various preservation temperatures and methods of preservation on post-transplant outcomes in renal grafts and other organ grafts. Drawing from preclinical, clinical, and meta-analytic studies, we compare hypothermic (0–4 °C), moderate hypothermic (10 °C), subnormothermic (20–32 °C), normothermic (35–37 °C), and subzero preservation strategies, and cellular and molecular changes that occur in renal grafts and other solid organ grafts during preservation at these temperatures. Overall, temperature-controlled machine perfusion outperforms static preservation of renal grafts and other solid organ grafts from marginal and deceased donors, potentially expanding donor pools and improving long-term graft survival, and suggests the need for future research to determine optimal preservation temperature for renal grafts and other solid organ grafts to improve viability and post-transplant outcomes.

## 1. Introduction

Kidney transplantation is the preferred therapeutic intervention for patients with end-stage renal disease, as it provides transplant recipients with a better quality of life and a significant survival advantage at a relatively cheaper cost compared to dialysis therapy. However, the global donor kidney shortage crisis presents significant healthcare challenges, as the available donor kidneys do not meet the high demand for kidney transplantation [1]. According to national statistics, a record number of 27,759 kidney transplants were performed at the end of 2024 in the United States. This figure represents a 1.6% increase compared to 2023 [2]. Despite this significant increase, the kidney transplant waitlist continues to grow significantly, with an increasing number of deaths while waiting. A major challenge in kidney transplantation is an unavoidable pathological condition referred to as ischemia–reperfusion injury (IRI) during procurement, storage, and upon transplantation of the renal graft. IRI can lead to delayed graft function (DGF) or primary non-function (PNF), which further complicates the health of transplant recipients and increases the risk of graft rejection [3]. 

### Methods of Preservation of Renal Grafts

The degree of IRI is partly attributable to renal graft storage duration and method. There are two primary preservation methods implemented to mitigate IRI: static cold storage (SCS) and machine perfusion (MP). The choice of preservation method as well as preservation temperature plays a critical role in determining post-transplant outcomes. SCS has been the clinical gold standard for preservation of renal grafts for over 50 years due to its simplicity and cost-effectiveness [4,5]. It involves graft preservation in a storage solution at 4 °C on ice to reduce metabolic demand and slow enzymatic degradation within the graft. Despite its simplicity and cost-effectiveness, which make SCS widely used across different transplant centers, prolonged graft preservation by SCS leads to cold ischemic injury, which is exacerbated upon reperfusion [6,7,8]. This implies that very limited period donor organs can withstand before prolonged cold ischemic time (CIT) begins to reduce graft viability, as well as an increased risk of IRI and post-transplant complications [9]. Moreover, SCS does not allow assessment of graft function, viability, and the possibility of graft repair before transplantation. Furthermore, several studies revealed discrepancies in the actual storage temperature during SCS. These studies highlighted that the temperatures on the surface and within renal grafts during SCS are close to 0 °C rather than the expected 4 °C [10,11]. To meet the increasing demand for donor kidneys, utilization of marginal kidneys from deceased donors has risen considerably, thus expanding the donor pool. Consequently, MP has gained widespread adoption due to its demonstrated superiority over SCS in preserving suboptimal renal grafts and other solid organ grafts. The development of MP systems allows for the control of preservation temperatures as well as oxygenation required for increased metabolic demand at near physiological temperatures. MP provides a dynamic preservation technique by continuously perfusing the donor organ with preservation solutions while flushing out toxic metabolites at varying temperatures [12]. This preservation method enables functional assessment and therapeutic interventions of renal grafts and serves as a graft repair platform during preservation and thus could be used to improve the quality of organs from marginal and deceased donors, and extend the acceptable organ preservation time [13,14,15]. Various MP techniques such as hypothermic machine perfusion (HMP; 4 °C), hypothermic oxygenated machine perfusion (HOPE; 4 °C), normothermic machine perfusion (NMP; 35–37 °C) and subnormothermic machine perfusion (SNMP; 20–32 °C) have been reported in several studies to produce superior outcomes compared to SCS, and have recently gained attention as alternatives to the conventional SCS of renal grafts [16,17,18,19]. In this review, we evaluated cellular and molecular effects of different preservation temperatures on metabolic activity, IRI, and post-transplant outcomes of donor kidneys and other solid organ grafts during SCS and MP, with the aim of highlighting emerging evidence supporting temperature-optimized preservation strategies and their potential to improve graft survival and patient outcomes.

## 2. Ischemia–Reperfusion Injury and Its Impact on Renal Grafts

IRI is an inevitable consequence of solid organ transplantation, arising from the temporal cessation of blood flow and oxygen deprivation during organ procurement, preservation, and transportation (ischemia) followed by restoration of blood circulation and oxygen (reperfusion) upon transplantation. IRI significantly impairs early graft function and long-term renal graft survival by activating inflammatory pathways, triggering cell death, and renal graft dysfunction [20]. Additionally, suboptimal kidneys from marginal and deceased donors are particularly vulnerable to prolonged cold ischemic time (CIT), which considerably increases the risk of IRI-related post-transplant complications. This increased susceptibility results in reduced cell viability, mitochondrial impairment, vascular damage, inflammation, graft dysfunction, and a high rate of donor kidney decline or discard [21,22,23,24]. Therefore, there is an urgent need to fully understand IRI at the cellular and molecular levels to determine the optimal preservation temperature and develop targeted treatment options that will enhance graft function, reduce post-transplant complications, and prolong the survival of transplanted kidneys and recipient patients.

### Molecular Mechanisms Underlying Renal IRI

**Figure 1:**
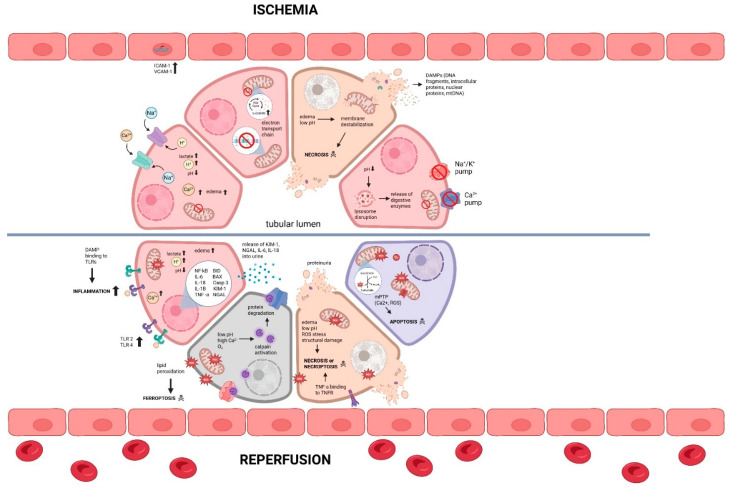
Molecular mechanisms underlying renal graft damage during ischemia–reperfusion injury. Pathological changes during ischemia of the renal graft are represented by the top panel, while the bottom panel depicts the subsequent molecular mechanisms during reperfusion. Abbreviations: Calcium ion (Ca^2+^), Damage-associated molecular patterns (DAMPs), deoxyribonucleic acid (DNA), Hydrogen ion (H^+^), kidney injury molecule-1 (KIM-1), mitochondrial DNA (mtDNA), mitochondrial permeability transition pore (mPTP), neutrophil gelatinase-associated lipocalin (NGAL), reactive oxygen species (ROS), sodium ion (Na^+^), Toll-like receptors (TLRs), Toll-like receptor 2 (TLR2), Toll-like receptor 4 (TLR4), tumor necrosis factor-alpha (TNF-α), and tumor necrosis factor receptor (TNFR). Created in BioRender. Ortas, T. (2025). https://app.biorender.com/illustrations/689c96ec4fef9b09d4c5ed5e (accessed on 25 January 2026).

IRI in renal grafts involves a complex of pathophysiological processes involving the activation of cell death pathways, transcription reprogramming, microvascular damage, and activation of the innate and adaptive immune system [25]. Multiple pathways and signaling cascades are involved in IRI, making it a critical area of study for improving the viability and longevity of marginal organs in transplantation. Oxygen is the single most important substrate necessary for cellular energy production, without which cells are forced to switch to anaerobic respiration [26]. Renal ischemia, as observed in hypothermic preservation of renal grafts and illustrated in Figure 1, creates a hypoxic environment, causing a shift from mitochondrial respiration to anaerobic metabolism, with many downstream effects, including reduced production of adenosine triphosphate (ATP), lactic acid build-up, and the alteration in intracellular ionic environment [27,28,29]. This metabolic shift disrupts cellular homeostasis, causing a decrease in pH (intracellular acidosis), Na^+^/K^+^ pump failure, intracellular Ca^2+^ overload, and intracellular leakage of lysosomal enzymes, all of which create a toxic environment in the cell, leading to cellular edema, rupture, and necrotic cell death [30,31] (Figure 1).

Restoration of blood supply and oxygen upon reperfusion causes increased mitochondrial production of reactive oxygen species (ROS; a destructive mediator of cell death and tissue injury), which overwhelms the antioxidant capacity of renal cells, leading to oxidative stress and ultimately oxidative damage of lipids, proteins, and DNA [32,33]. Reperfusion also causes further increase in Ca^2+^ overload, and triggers the formation and opening of mitochondrial permeability transition pores (mPTP; calcium-dependent channels formed in mitochondrial inner membrane under pathological conditions), causing release of apoptosis-inducing factors and other cell death factors, leading to cell apoptosis and other forms of regulated cell death such as necroptosis and ferroptosis [34,35] (Figure 1). The increase in ROS production and the consequent oxidative stress during reperfusion trigger activation of Toll-like receptors (e.g., TLR2 and TLR4) acting as sensors of pathogen-associated molecular patterns (PAMPs) and damage-associated molecular patterns (DAMPs), which activate both the innate and adaptive immune system, leading to inflammation and renal graft injury through increased production and release of pro-inflammatory cytokines such as interleukin-1beta (IL-1β), IL-6, tumor necrosis factor-alpha (TNF-α) [36] (Figure 1). Paradoxically, reperfusion induces microvascular injury, characterized by endothelial cell swelling, increased vascular permeability, and sustained immune activation, leading to microvascular disturbances and renal graft thrombosis [37]. Interestingly, the severity of renal IRI is closely linked to the renal graft preservation temperature, with evidence suggesting that warmer preservation temperatures may mitigate mitochondrial dysfunction, oxidative stress, and endothelial damage compared to hypothermic storage at 4 °C [38,39]. Therefore, optimizing renal graft preservation temperature to minimize renal IRI is crucial for improving graft quality and function and post-transplant outcomes. The storage methods and temperatures used across graft types and comparator models in the cited studies are summarized in Table 1.

## 3. Cellular and Molecular Changes in Organ Grafts During Hypothermic Preservation at 4 °C Temperature

**Figure 2:**
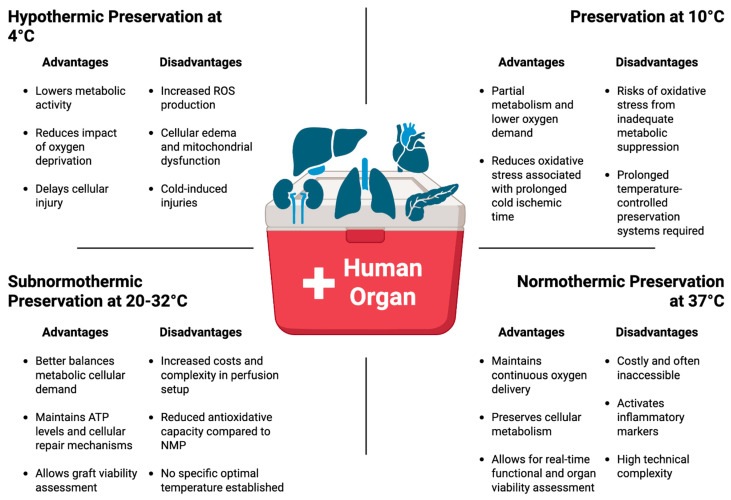
Summary of the impact of various preservation temperatures and methods of preservation on donor organs. The various organ preservation temperatures and methods of preservation have benefits and drawbacks that impact organ quality and function, and ultimately post-transplant outcomes. Created in BioRender. England, C. (2026) https://BioRender.com/rxu6e46 (accessed on 25 January 2026).

Temperature regulation during organ preservation is crucial because it directly regulates metabolic and enzymatic activities, ATP production, oxidative stress, and IRI-related post-transplant complications [73,74]. As illustrated in Figure 2, organ preservation at 4 °C significantly lowers metabolic activities, including a reduction in adenosine triphosphatases (ATPases) energy consumption via ion-balancing, reduces the impact of oxygen deprivation, preserves transmembrane electrochemical gradients, temporarily inhibits activation of apoptotic biochemical pathways, and delays cellular injury [75]. Without reducing ATP and oxygen consumption, the hypoxic period seen in organs during hypothermic preservation inhibits ion exchange via ATPases, leading to cell membrane depolarization and resulting in cellular swelling and eventual cell death [75,76]. According to van’t Hoff’s equation involving changes in temperature relating to standard enthalpy change in a chemical reaction, exposure to cold temperatures causes a reduction in metabolic activity by approximately 50% for every 10 °C drop in temperature. This means that the rate of cellular reactions at 4 °C would be approximately 40% as effective and 90% slower than cellular reactions at 37 °C [26,74,77]. This reduction in metabolic activity and consequent reduction in oxygen demand are the primary reasons for 4 °C being the current clinical standard temperature for graft preservation. Also, the use of storage solutions during hypothermic preservation provides additional protection against cellular edema, as these solutions contain a balanced mix of ions (Na^+^ and K^+^), buffers, and osmotic agents to mimic and maintain the intracellular or extracellular environment upon cold-induced dysfunction of Na^+^/K^+^-ATPase pumps [78]. Furthermore, SCS or HMP prevents lysis of organelles such as lysosomes, which can release autolytic enzymes that cause cell death [79]. Although the metabolic demand of grafts is significantly reduced at hypothermic temperatures, prolonged cold preservation leads to membrane damage, vascular endothelial cell injury, inflammation, mitochondrial impairment, cell death, and ultimately graft dysfunction [7,80]. Exposure to near freezing temperatures can induce additional cellular damage, including protein denaturation, mitochondrial edema due to the formation of ice crystals within the cells [81]. Interestingly, a relatively new area of research is examining the use of subzero preservation temperatures without the formation of ice crystals to extend CIT with minimal structural and viability changes.

While HMP is similar to SCS in terms of suppressing cellular metabolic function, it differs in that it mimics physiological vascular perfusion and facilitates the excretion of metabolic by-products with the organ [27,82]. By continuously circulating preservation solution through the organ at cold temperature, HMP flushes the microvasculature of toxic metabolites as well as reduces the production of free radicals [83]. Similarly to HMP, HOPE utilizes continuous perfusion to facilitate the removal of harmful metabolic buildup but differentiates itself by perfusing the graft with a cold, oxygen-saturated solution; thus, mimicking a physiological environment and preventing toxic metabolic waste products, making more suboptimal organs usable and reducing the rate of donor organ discard. Organ preservation by HOPE has been reported to reduce cytokine release and improve organ function [84,85].

### 3.1. Hypothermic Preservation of Renal Grafts at 4 °C Temperature

Since the 1970s, SCS has been the standard method of preserving renal grafts for transplantation and has been used by our research team and other research groups in experimental models of kidney transplantation [6,51,86,87,88]. However, research has suggested that the use of HMP or HOPE during preservation of marginal and deceased donor organs has led to better post-transplant clinical outcomes [42,75]. In a retrospective matched-pairs analysis, renal grafts preserved with HMP demonstrated significantly lower rates of DGF compared to those preserved with SCS (29.8% vs. 36.1%, *p* < 0.001), and adjusted analyses indicated a potential protective effect of HMP against DGF [89]. Similarly, HMP significantly reduced the risk of DGF in renal grafts compared to SCS in a meta-analysis (95% CI: 0.67 to 0.90; *p* = 0.0006) [40]. While less research has been performed to investigate the impact of HOPE vs. SCS preservation compared to HMP vs. SCS on renal grafts, there are studies that have demonstrated the potential of HOPE to protect against immune activation and improve early graft function [41,85]. In an animal study comparing SCS vs. HOPE, HOPE-treated renal grafts experienced significantly improved early graft function compared to SCS-treated grafts (*p* < 0.0001) [41]. Similarly, a randomized, double-blind, paired clinical trial involving renal grafts from deceased donors found that graft preservation by HOPE showed significantly less severe complications (11% vs. 16%; *p* = 0.032) and less incidence of graft failure (3% vs. 10%; *p* = 0.028) compared to renal grafts treated and preserved by HMP [42]. However, only 106 kidney pairs were ultimately transplanted from the original cohort of 197 randomized pairs, underscoring the limited sample size and highlighting a key limitation of available clinical evidence directly comparing HOPE and HMP. Despite these constraints, the favorable clinical outcomes associated with both preservation strategies have driven continued investigation into their use as tools to expand the pool of transplantable donor kidneys, particularly those from DCD or ECD that were historically discarded due to poorer expected outcomes.

### 3.2. Hypothermic Preservation of Non-Renal Grafts at 4 °C Temperature

#### 3.2.1. Liver Graft Preservation at 4 °C Temperature

Similarly to kidney transplantation, liver graft preservation by HMP and HOPE has shown great promise, with better clinical outcomes than SCS. In a systemic review and meta-analysis of liver graft outcomes after preservation by SCS and HMP, HMP-treated grafts were associated with significantly less complications (95% CI 0.46–0.84; *p* < 0.01) and non-anastomotic biliary strictures (NAS) (95% CI 0.19–0.61; *p* = 0.0003), in addition to significantly increased one-year graft survival (95% CI 1.54–3.45; *p* < 0.01) and 1-year patient survival (95% CI 1.15–2.79; *p* = 0.01) compared to SCS-treated grafts [43]. Similarly, Tang et al. [44] reported in a systematic meta-analysis that HOPE-treated liver grafts had a significantly reduced risk of post-operative total biliary complications (95% CI 0.61–0.91; *p* = 0.004) as well as a reduced risk of NAS (95% CI 0.26–0.70; *p* < 0.01) compared to SCS-treated grafts. Additionally, HOPE has been suggested for the preservation of liver grafts from deceased donors. A randomized clinical trial using liver grafts from deceased donors reported decreased incidence of NAS by about two-thirds in HOPE-treated grafts compared to the SCS group (95% CI 0.14–0.94; *p* = 0.03) [45]. Recent unpublished data presented at the World Transplant Congress using the Paragonix LIVERguard system demonstrated that livers preserved under controlled hypothermic conditions achieved safely extended cold ischemic times and exhibited a 51% reduction in early allograft dysfunction compared with livers preserved by conventional static cold storage [90]. These findings suggest that precise, static control of preservation temperature, even in the absence of active perfusion, may substantially improve liver preservation quality. Such an approach has the potential to enhance graft viability, reduce early post-transplant complications, and increase the utilization of donor livers by mitigating the limitations associated with traditional cold storage.

#### 3.2.2. Cardiac Graft Preservation at 4 °C Temperature

Static controlled hypothermic preservation of donor hearts has become increasingly adopted as an alternative to conventional static cold storage on ice, primarily to avoid exposure to potentially harmful freezing temperatures. Analysis of the GUARDIAN-Heart Registry demonstrated that controlled hypothermic preservation using the SherpaPak™ Cardiac Transport System was associated with a significant reduction in severe primary graft dysfunction as well as lower 2-year mortality rates compared with hearts preserved using traditional on-ice SCS [46]. These findings underline the critical importance of precise temperature control during static preservation, highlighting that avoidance of subzero and near-freezing temperatures may substantially improve cardiac graft outcomes. However, unlike kidney and liver transplantation, the impact of HMP and HOPE in heart transplantation has not been studied extensively. In a study performed by Andrijauskaite et al. [47], it was observed that HMP-treated human hearts experienced significantly more ventricular relaxation (*p* < 0.05) and downregulated expression of inflammatory markers compared to SCS-treated cardiac grafts. Clinical evidence further supports the feasibility of this approach. Results from a multicenter Australian and New Zealand experience with HOPE-preserved donor hearts demonstrated procedural safety, 100% patient survival at 30 days, and successful extension of preservation times to up to 9 h [48]. In addition, a recent case report described successful transplantation of a single donor heart following a prolonged cold ischemic time of 12 h using HOPE, with immediate post-transplant graft function and extubation of the recipient within 10 h after surgery [91]. Overall, although available data remain limited, emerging evidence suggests that machine perfusion strategies, including both HMP and HOPE, may safely extend cold ischemic time in heart transplantation. This has important implications for expanding the donor pool, improving organ utilization, and overcoming logistical constraints associated with long-distance organ procurement.

#### 3.2.3. Lung Graft Preservation at 4 °C Temperature

Cold preservation temperatures also impose strict limitations on allowable preservation time in lung transplantation, with cold ischemic times traditionally restricted to approximately 6 h to minimize the risk of post-transplant complications. In a retrospective analysis evaluating controlled hypothermic preservation of donor lungs with extended cold ischemic times of up to 15 h, 4 of 13 recipients developed grade 3 primary graft dysfunction within the first 72 h following transplantation; however, graft function recovered by 72 h in all affected cases [92]. In this cohort, four patients required postoperative extracorporeal membrane oxygenation (ECMO) support [92]. Although these findings indicate an increased incidence of early graft dysfunction with prolonged preservation, they also suggest that controlled hypothermic temperature management may permit safe extension of lung preservation time, with reversible early injury and acceptable short-term outcomes. Similarly to heart transplantation, very little research has been performed on the impact of HMP and HOPE at 4 °C on lung transplantation. In a canine model of donation-after-cardiac-death lung transplantation, the use of HMP resulted in significantly greater lung oxygenation and dynamic pulmonary compliance (*p* < 0.01) along with markedly reduced severity of pulmonary edema (*p* < 0.05) compared to the SCS group [49]. However, further studies from other research groups are required to accentuate this promising experimental result and the underlying mechanisms at the molecular level before moving to human clinical trials to extend cold preservation time.

#### 3.2.4. Pancreatic Graft Preservation at 4 °C Temperature

Much less research into the impact of HMP and HOPE compared to SCS in pancreas transplantation has been conducted compared to studies involving kidney or liver transplantation. Due to the poor results initially encountered when treating pancreas grafts with HMP, there was a decline in research for the use of HMP in whole pancreas transplantation, although the encouraging results in the use of HMP and HOPE in liver and kidney transplantation have aroused the interest of some researchers to revisit the use of HMP and HOPE in pancreas transplantation [93]. In a clinical study comparing the impacts of HOPE and SCS on pancreatic grafts procured from deceased donors, Leemkuil and colleagues [50] reported that HOPE-treated grafts showed significantly improved viability and higher ATP concentration (*p* < 0.05) compared to SCS-treated grafts. Additionally, HMP rescued pancreata procured from deceased donors, which otherwise would have been discarded, to be used for clinical islet cell isolation [94]. Considering that HOPE outperforms HMP in studies performed so far, the use of HMP to rescue pancreata suggests the potential for the use of HOPE in pancreas transplantation in the future.

## 4. Cellular and Molecular Changes in Organ Grafts During Moderate Hypothermic Preservation at 10 °C Temperature

Over the past five years, preclinical and clinical studies have shown a growing interest in exploring moderate hypothermic preservation temperatures such as 10 °C to better balance the metabolic cellular demand and reduce the oxidative stress associated with prolonged cold ischemic storage in organ transplantation [95]. Preservation of organs at a 10 °C temperature provides slightly higher cellular metabolism that allows higher ATP production, reduces mitochondrial injury, and enhances mitochondrial antioxidant activities while reducing pro-inflammatory cytokines compared to traditional 4 °C preservation [55]. The increase in ATP and mitochondrial metabolism leads to increased function of the Na^+^/K^+^-ATPase pump, resulting in higher oxygen consumption and reducing cellular edema [49]. Furthermore, experimental organ preservation at 10 °C was associated with increased expression of anti-oxidative metabolites as well as decreased protein levels of cell-free mitochondrial DNA (mtDNA) and pro-inflammatory cytokines [96]. These early results highlight the cytoprotective molecular mechanisms at 10 °C that preserved organs by slightly increasing cell metabolism and antioxidant defense mechanisms without activating inflammatory pathways. This allows for the prolongation of CIT in most solid organ transplants (Figure 2). Moreover, preservation at 10 °C further avoids the severe metabolic demand and complexity required at subnormothermic and normothermic preservations. It is worth noting that most organ preservation at 10 °C involves the use of a controlled hypothermic cooler system to maintain temperatures in a static approach with accuracy and cost-effectiveness, enabling longer CIT.

### 4.1. Renal Graft Preservation at 10 °C Temperature

Previous research has demonstrated promising strategies for pretreating donor kidneys to improve post-transplant outcomes and attenuate injury associated with cold preservation. These approaches have included the use of anti-inflammatory and antioxidant agents, such as corticosteroids, dopamine, carbon monoxide, and hydrogen sulfide-based gasotransmitters, administered during preservation at 4 °C [97,98,99,100]. More recently, our research team has shown that applying such pretreatment interventions at 10 °C may provide a synergistic advantage, as partially preserved metabolic activity at this temperature supports endogenous cellular protective and repair mechanisms. In an in vitro hypoxia–reoxygenation model, we demonstrated that preservation of rat proximal tubular epithelial cells at 10 °C in the presence of sodium thiosulfate (STS)—a clinically approved H_2_S donor—significantly improved cell survival compared with conventional cold preservation strategies [51]. Hydrogen sulfide is an endogenously produced gaseous signaling molecule that has anti-inflammatory, anti-apoptotic, anti-fibrotic, and antioxidant properties, making it a promising candidate for therapeutic intervention in renal IRI [101]. Furthermore, 24 h of SCS of renal grafts in STS-supplemented preservation solution at 10 °C improved early graft function and organ viability, reduced kidney injury markers and inflammation, and prolonged survival of recipient rats in a murine model of syngeneic kidney transplantation [51]. A similar result was reported in another rat model of kidney transplantation in which renal grafts stored at 10 °C showed less apoptosis and necrosis compared to storage at 4 °C and reduced mitochondrial injury and graft dysfunction after reperfusion [52]. These findings support the concept that combining temperature optimization with targeted cytoprotective therapies may represent a novel and effective approach to improving renal graft preservation and post-transplant function. However, further investigation is required to validate the efficacy of 10 °C renal graft preservation in large animal models and in marginal donor kidneys, in order to expand our understanding of its translational potential and define its role in clinical kidney transplantation.

### 4.2. Non-Renal Graft Preservation at 10 °C Temperature

Apart from kidneys, the impact of moderate hypothermic preservation at 10 °C temperature is currently being investigated in other solid organs and is so far showing great clinical promise.

#### 4.2.1. Liver Graft Preservation at 10 °C Temperature

Liver preservation at 10 °C has been investigated in SCS and in HOPE to increase the utilization of liver grafts from deceased donors, which are associated with increased risks of IRI-induced post-transplant complications. A recent preclinical study of oxygenated static liver preservation at 10 °C using a temperature-adjustable refrigerator demonstrated that liver grafts from deceased donor pigs had significantly improved vascular viability, bile production, biliary biomarkers, and a decrease in DNA damage compared to SCS at 4 °C [53]. Moreover, untargeted metabolomics showed improved mitochondrial health by enhanced electron transport chain function while sustaining aerobic respiration in livers stored at 10 °C compared to storage at 4 °C [53]. Results from this study highlight a novel approach to mitigate IRI in liver grafts from deceased donors and improve liver function in a simple and cost-effective way, which can extend CIT and expand the pool of donor livers.

Another approach to mitigate IRI in liver transplantation is preservation by HOPE. Within 2 h of HOPE treatment at 10 °C, mitochondrial reprogramming occurs, allowing the recovery of ATP production and metabolism of anti-oxidative metabolites, preventing the production of ROS during reperfusion [54,102]. Therefore, graft preservation by HOPE at 10 °C reduces inflammation and prevents future injuries, allowing healthy liver graft recovery. In a multicenter-controlled trial involving liver grafts from deceased donors in which SCS 4 °C and HOPE at 10 °C were compared, 160 patients were recruited and equally enrolled into both groups. The incidence of non-anastomotic biliary strictures, a major complication in liver transplantation, was lower in the HOPE group (6 vs. 18%) as well as early graft dysfunction (26 vs. 40%) compared to the SCS group [45]. Moreover, a recent study investigated the safety and feasibility of prolonged use of HOPE at 10 °C (>4 h) rather than conventional 1–2 h of HOPE in donation-after-brain-death liver grafts, in which the authors observed no difference in adverse events, liver-related post-transplant complications, and graft survival between the two HOPE-treated groups [103]. Additionally, there was no difference in liver function markers and markers of IRI-induced oxidative stress in both groups [103]. Collectively, these studies suggest that utilization of HOPE in liver grafts from deceased donors is safe and allows for improved graft allocation; thus, decreasing the rate of discards of liver grafts from deceased donors.

#### 4.2.2. Lung Graft Preservation at 10 °C Temperature

Preservation of lung grafts at 10 °C has been extensively investigated in recent years in preclinical and clinical lung transplant settings. A major obstacle in lung transplantation is the currently accepted short CIT of 6–8 h. Therefore, extending the CIT of lung grafts is being extensively investigated, with one being a 10 °C preservation temperature. In a porcine model of lung transplantation, graft preservation by SCS at 10 °C for 36 h followed by 12 h of normothermic ex vivo lung perfusion resulted in significantly improved lung functional parameters and markedly enhanced mitochondrial antioxidant metabolites, including itaconate, glutamine, and N-acetylglutamine, compared to the 4 °C group [55,96]. Additionally, lung graft preservation at 10 °C preserved mitochondrial integrity, reduced the levels of lactate and calcium, as well as apoptosis, necrosis, and pro-inflammatory cytokines such as IL-1 and IL-8 [55]. These findings were further validated in an injured porcine lung transplant model, where graft preservation at 10 °C for 12 h resulted in higher dynamic lung compliances, increased production of cytoprotective metabolites within the graft while significantly reducing peak airway pressures, circulating cell-free mitochondrial DNA within the recipient plasma, and apoptosis relative to lungs undergoing immediate transplant [56]. These empirical findings allowed for the translation of 10 °C preservation of lung grafts into clinical settings with successful extension of CIT from 6 h to 24 h without any major post-transplant complications [55,104]. Most importantly, SCS of lung grafts at 10 °C reduces the incidence of grade 3 primary graft dysfunction at 72 h when compared to SCS at 4 °C (5.7 vs. 9.3%) [55,105]. Moreover, lung grafts preserved at 10 °C were not associated with prolonged mechanical ventilation, ICU time, longer hospital stays, and mortality rates up to 1 year follow-up [104]. Thus, preservation of lung grafts at moderate hypothermic temperature of 10 °C does not only improve donor lung acceptance rates and immunological matching between donors and recipients, but also transitions lung transplantation from an urgent procedure into a planned semi-elective surgery, and therefore, could become the standard of care for prolonged lung graft storage which would benefit transplant recipients as well as the team of healthcare professionals.

#### 4.2.3. Cardiac Graft Preservation at 10 °C Temperature

As with other organ grafts, preservation at 10 °C has shown promise in heart transplantation. Clinically, cardiac grafts are preserved for only 4–6 h on ice at 4 °C, as prolonged CIT often leads to edema, mitochondrial impairment, and graft dysfunction, and ultimately a higher rate of primary graft dysfunction and mortality of heart transplant recipients [106,107,108]. In a recent clinical study that examined preservation of 52 cardiac grafts at 10 °C in a controlled cooler and 156 cardiac grafts on ice at 4 °C, there was no significant difference in the rate of primary graft dysfunction, ICU time, and length of hospital stay between both groups [57]. However, there was a significant decrease in lactate levels in cardiac grafts preserved at 10 °C compared to 4 °C (3.6 vs. 5.1 mmol/L, *p* = 0.0016). Additionally, 44.2% of the grafts preserved at 10 °C with longer CIT (>4 h) showed better cardiac health compared to those preserved at 4 °C [57], suggesting safe extension of CIT in heart transplantation. Furthermore, a case study of cardiac graft preservation at 10 °C for over 10 h showed excellent early graft function without the need for mechanical circulatory support [109]. Early results of preservation of cardiac grafts at 10 °C are promising, and highlight the need to prolong CIT to allow improved donor–recipient matching and to increase the acceptance rate of donor hearts.

#### 4.2.4. Pancreatic Graft Preservation at 10 °C Temperature

The optimal CIT of pancreatic grafts associated with the best post-transplant outcome is limited to 12 h [110]. Since the complex vascular anatomy of the pancreas requires a low perfusion flow, utilizing the optimal perfusion parameters of flow and pressure is difficult to establish in machine perfusion systems. While high perfusion pressure can result in endothelial damage and an increased risk of thrombosis, low pressures can cause insufficient oxygenation [111]. Preservation of canine pancreatic grafts by HMP was initially shown to experience edema and tissue injury compared to SCS [112]. Twenty-five years later, this results contradicted findings from two studies using pancreatic grafts from human deceased donors in which HMP preserved pancreata, with higher ATP production without any evidence of edema [113,114]. Since 10 °C storage of other organs is gradually entering the clinical setting, preservation of pancreas at this moderate hypothermic temperature is an area of great interest, which can mitigate the challenges associated with 4 °C storage, and safely extend the accepted CIT in pancreas transplantation.

## 5. Cellular and Molecular Changes in Organ Grafts During Normothermic Machine Perfusion

Normothermic machine perfusion (NMP) at physiological temperatures of 35–37 °C has emerged as a valuable organ preservation strategy. As summarized in Figure 2, NMP maintains aerobic metabolism and prevents cellular and mitochondrial impairment that develops during cold ischemia. One of the key advantages of NMP is its ability to reduce the risk of post-transplant IRI by preventing the abrupt metabolic shift that occurs when an oxidatively stressed hypothermically preserved organ graft is suddenly reintroduced to warm blood circulation [115]. By maintaining oxygen delivery, NMP sustains mitochondrial function, minimizes oxidative stress, and improves endothelial integrity, which collectively enhances graft viability [116]. Another important benefit of NMP is that it allows for real-time graft viability and functional assessments before transplantation. The ability to assess graft viability in real-time during NMP has been particularly beneficial in marginal donor organs to help clinicians predict functionality and viability before complete rejection and discard of donor organs [117]. This is very important, especially in marginal donor organs due to their increased susceptibility to prolonged CIT. Additionally, NMP has been shown to prolong preservation time beyond the limits of SCS, facilitating logistics and expanding the donor pool by allowing transplantation over longer distances.

In renal grafts, for example, the continuous oxygen and nutrient delivery through NMP maintains oxidative phosphorylation and supports ATP replenishment, which enables functional activity such as urine production and hormone secretion in donor kidneys [118], bile production, ATP synthesis, lactate clearance, and biochemical markers of hepatocellular injury (for liver grafts) [119,120,121], coronary flow and lactate clearance (for cardiac grafts) [122], gas exchange and pulmonary vascular resistance (for lung grafts) [123], and insulin secretion (for pancreatic grafts) [15,124] before transplantation. In pancreas transplantation, for example, NMP in porcine models has shown reduced apoptosis and oxidative DNA damage compared to SCS [124]. Despite these prominent advantages, the NMP setup is technically complex and challenging due to availability, as it requires strict control of oxygen delivery and perfusion pressure, along with a constant heating system, tight pH and glucose regulation, red blood cell hemolysis, and risk of infection and immunization [125,126]. Also, NMP involves an expensive blood-based perfusion system, and disturbances to perfusion at warmer temperatures place organ grafts at a higher risk of inflammation and failure after transplantation. Furthermore, a failure of the perfusion system would immediately lead to graft loss [125,127]. These disadvantages limit global adoption of NMP. Nonetheless, when optimized, NMP provides a physiologically favorable environment that improves graft preservation and enables real-time functional assessment, which allows for better utilization of satisfactory and marginal donor organs [117].

### 5.1. Renal Graft Preservation by Normothermic Machine Perfusion

NMP in human kidney transplantation has demonstrated some superior outcomes compared to SCS, including reduced incidence of DGF and improved graft survival, particularly in marginal donor kidneys [128]. NMP maintains renal grafts around 37 °C using oxygenated blood or perfusate, enabling near-normal metabolism ex vivo and facilitating functional assessment such as urine production and creatinine clearance [118]. While short-term NMP has proven feasible and safe, it has not yet demonstrated its complete superiority in clinical outcomes. A randomized clinical trial involving 338 human deceased donor kidneys showed no significant difference in the rate of DGF (~60%) between NMP and SCS groups (*p* = 0.62) [58]. Importantly, NMP has been confirmed to be safe without increasing thrombotic or infectious complications, and kidneys under NMP actively produced urine, hormones (renin, erythropoietin), and preserved ATP level [128,129]. Biomarkers observed during ex vivo NMP, such as stable urine pH and gradually rising perfusate lactate, aspartate aminotransferase, lactate dehydrogenase, and inflammatory cytokines, may aid in evaluating renal graft quality and predicting post-transplant outcomes. Ongoing studies seek optimal perfusion protocols to further expand the pool of donor kidneys and enhance long-term transplant outcomes.

### 5.2. Non-Renal Graft Preservation by Normothermic Machine Perfusion

#### 5.2.1. Liver Graft Preservation by NMP

NMP rapidly transitioned from experimental studies to clinical use, maintaining liver function ex vivo by providing warm oxygenated blood and nutrients, evidenced by bile production, ATP synthesis, and lactate clearance [119,120,121]. A landmark multi-center European trial demonstrated that continuous NMP reduced liver graft injury, decreasing peak aspartate aminotransferase by ~49% and incidence of early graft dysfunction (10% NMP vs. 30% SCS) [59]. Similarly, a U.S. trial (OCS Liver device) reported a significantly reduced rate of early graft dysfunction in NMP compared to traditional SCS (18% NMP vs. 31% SCS; *p* ≈ 0.01) [60]. However, another U.S. randomized trial with a different device found no significant difference in the rate of early graft dysfunction between NMP and SCS (20.6% vs. 23.7%, respectively), although post hoc analyses suggested greater benefits in higher-risk donors [61]. NMP also provides valuable real-time viability metrics such as perfusate lactate clearance and bile quality, enabling safe transplantation of previously discarded liver grafts (UK “VITTAL” study, >95% one-year survival) [120]. Currently, hybrid perfusion strategies combining cold and warm perfusion phases are being investigated to maximize the benefits of reduced ischemic injury and controlled inflammation.

#### 5.2.2. Lung Graft Preservation by NMP

Lungs are exceptionally vulnerable to ischemic injury from SCS, leading to edema, inflammation, and PGD. Ex vivo lung perfusion (EVLP) at normothermia has revolutionized lung transplantation by enabling functional assessment and reconditioning. Physiologically, EVLP preserves ATP levels, reduces inflammatory mediators, and improves lung compliance and oxygenation. Normothermic EVLP significantly reduces severe primary graft dysfunction (17.7% EVLP vs. 29.7% SCS; *p* = 0.015) and increases utilization of marginal donor lungs without compromising survival (INSPIRE trial) [62]. Long-term outcomes were compared in ECD donor lungs with SCS in the EXPAND trial, reporting similar survival and any bronchiolitis obliterans syndrome rates [130]. Overall, EVLP has successfully increased transplant volumes by enabling transplantation of initially unsuitable donor lungs, with outcomes comparable to conventional selection. Research continues into prolonged EVLP and adjunct therapies (e.g., antibiotics, gene therapy) to further enhance lung graft quality and functional viability.

#### 5.2.3. Cardiac Graft Preservation by NMP

Cardiac graft preservation by SCS is limited to approximately 4–6 h due to poor tolerance to cold ischemia. NMP systems (e.g., TransMedics OCS Heart) enable continuous coronary perfusion with warm oxygenated blood, significantly extending preservation time, maintaining myocardial ATP level, and enabling functional assessments such as lactate clearance and evaluation of myocardial contractility [131]. Clinical trials demonstrated the effectiveness of NMP in enabling the successful transplantation of cardiac grafts from deceased donors, significantly expanding the donor pool [63]. A UK study showed equivalent one-year survival rates (~91% DCD vs. 89% DBD) using NMP or normothermic regional perfusion (NRP) [132]. Interestingly, a non-randomized U.S. study found no significant difference between NMP and SCS in terms of rejection rate, cardiac allograft vasculopathy incidence, and survival rate [64]. This suggests that further studies evaluate long-term outcomes, inflammatory profiles, and potential reduction in reperfusion injury markers in NMP.

#### 5.2.4. Pancreatic Graft Preservation by NMP

Recent proof-of-concept studies demonstrate that NMP can preserve pancreas viability and metabolic function ex vivo. In a 2023 study, six discarded human pancreata maintained metabolic activity, insulin secretion, and minimal histological injury during a 4 h NMP [133]. Although early experimental results are promising, clinical validation is pending. Future research will clarify the potential benefits of NMP in improving transplant outcomes, such as reduced pancreatitis, thrombosis, and improved long-term graft survival.

## 6. Cellular and Molecular Changes in Organ Grafts During Subnormothermic Machine Perfusion

SNMP at 20–32 °C supports partial metabolism with lower oxygen demand while maintaining ATP levels and improving cellular repair mechanisms, potentially reducing the severity of IRI-induced post-transplant complications [127] (Figure 2). Preservation of human and rat liver grafts from marginal donors at SNMP temperatures has shown an increase in ATP content, oxygen uptake, bile production, mitochondrial activity, and microcirculatory health [127,134]. A similar beneficial outcome was observed in renal grafts, with improved kidney function, reduced IRI-related post-transplant complications, and safely extended the duration of graft preservation in porcine models of donation-after-cardiac-death kidney transplantation [66,129]. SNMP is relatively a simpler protective method of preservation for marginal organs, and further clinical investigations are required to expand the pool of available donor organs. At SNMP temperatures, continuous oxygenation with an oxygen carrier is required to sustain the increased metabolic demand and energy production. Unlike moderate hypothermic preservation at 10 °C, SNMP temperatures increase metabolic activity, but not to the point where an oxygen carrier perfusate is necessary. Preservation at SNMP temperatures increases energetic cofactors and tricarboxylic acid cycle intermediates needed for ATP production. Metabolic study of liver grafts preserved by SNMP shows an increased level of nicotinamide adenine dinucleotide phosphate (NADPH; a crucial coenzyme that fuels cellular biosynthesis), which highlights increased ATP levels and may contribute to improved oxygen radical scavenging ability by increasing the levels of glutathione [135]. Therefore, maintaining preservation at subnormothermia increases active cellular metabolism, protecting IRI, and permits rejuvenation and repair of suboptimal organs from marginal donors while allowing for organ functional testing and viability assessment without activating immune pathways.

Organ graft preservation at subnormothermic temperatures offers some advantages over HMP and NMP. For example, oxygen delivery at intermediate temperatures in SNMP can be met with a simple acellular perfusate rather than the necessity to use blood as a perfusate at normothermic conditions [136]. This is very important in kidney transplantation because the ex vivo perfusion of red blood cells through renal grafts consistently leads to hemolysis, which results in free hemoglobin accumulation. The accumulated free hemoglobin can lead to heme toxicity that results in acute kidney injury [137]. Therefore, SNMP with acellular perfusate in kidney transplantation allows for an increased ex vivo preservation for transportation and viability assessments of donor kidneys.

### 6.1. Renal Graft Preservation by SNMP

A major obstacle in using blood-based perfusate in kidney transplantation is the hemolysis of red blood cells that leads to the accumulation of hemoglobin, causing acute kidney injury [138]. Therefore, only up to 6 h of perfusion with blood has been reported in human kidney transplantation. At subnormothermic temperatures, the metabolic and oxygen demand is met with a cell-free perfusate, thereby allowing for longer preservation time in kidney transplantation. In a porcine model of donation-after-cardiac-death kidney transplantation, renal grafts perfused at various warm temperatures with oxygenated blood show that SNMP perfusion resulted in the greatest reduction in apoptosis and kidney injury markers with maximum renal blood flow and urine output, highlighting that 21–22 °C perfusion is the optimal temperature to avoid hemolysis and blood clots for up to 4 h [65,139]. In an oxygenated acellular-based perfusate, prolonged perfusion of discarded human kidneys at 32 °C showed successful preservation for up to 12 h without any increase in kidney injury markers until after 24 h of preservation [140]. Another study demonstrated the success of prolonged perfusion of donation-after-circulatory-death kidneys in a porcine autotransplant model and in discarded human kidneys at 22–25 °C with an acellular-based perfusate for up to 24 h. The authors observed that the renal grafts exhibited significantly lower levels of serum creatinine and blood urea nitrogen post-transplantation for 7 days compared to SCS [66]. The acellular perfusate was based on culture media (DMEM/F-12) supplemented with human serum albumin, tricarboxylic acid cycle intermediates, and antibiotics, with continuous hemofiltration to remove metabolic toxins and maintain homeostasis. Interestingly, a more recent study demonstrated that discarded human kidneys can be perfused at 25 °C for at least 4 days while maintaining metabolic, functional, and structural integrity [141]. Furthermore, our group previously explored the efficacy of SNMP preservation (21 °C) supplemented with a hydrogen sulfide donor (AP39) as a therapeutic intervention for renal IRI, which showed a significant reduction in apoptotic injuries and an overall increase in renal functions, validating the use of therapeutic interventions in SNMP preservation [139]. Overall, these recent studies and advancements in perfusion setups are a major progress in the field of SNMP, and therefore, a steppingstone towards initial clinical trials.

### 6.2. Non-Renal Graft Preservation by SNMP

#### 6.2.1. Liver Graft Preservation by SNMP

Earlier studies involving SNMP of liver grafts have demonstrated its advantages over HMP with improved mitochondrial activity and cellular metabolism, as well as improved liver functions (bile, urea, and albumin production) [127,135]. Although recent metabolic studies have shown higher levels of ATP during SNMP, a significant decrease in glutathione levels was observed when compared to NMP [19,67]. These observations indicate a reduced antioxidant capability during SNMP, as glutathione is an important naturally occurring antioxidant that eliminates ROS within cells. Therefore, supplementation of antioxidative metabolites is a potential therapeutic intervention to further expand the utilization of SNMP in liver transplantation. Furthermore, a more recent study demonstrated the feasibility of prolonged preservation of discarded human livers for 24 h with improved liver parameters during SNMP [142]. Although heterogeneity in liver groups is present in this study, modification of the protocol could offer a long-term storage of suboptimal liver grafts until transplantation.

#### 6.2.2. Lung Graft Preservation by SNMP

Currently, all ex vivo lung perfusion (EVLP) protocols use NMP to accurately assess lung graft function and viability. Due to increased inflammatory cytokine production associated with NMP, some studies have investigated the use of EVLP in SNMP in lung transplantation. However, these studies are limited to rat models of donation-after-cardiac-death lung transplantation. EVLP of rat lungs at 28 °C has shown a significant improvement in lung function, oxygenation, higher ATP levels, and a reduction in the levels of pro-inflammatory cytokines and chemokines [68,143,144,145]. A more recent study validated the reduced inflammatory cytokine environment, which accompanied the decreased lung injury in SNMP compared to NMP, suggesting that these effects are due to decreased glycolysis metabolism through upregulated expression of mTORC, HIF-1α, and NLRP3 at normothermic temperatures [146]. However, subnormothermic EVLP is still in its early stages, and additional validation is required in larger animals to support its clinical translation.

#### 6.2.3. Cardiac Graft Preservation by SNMP

Preservation of cardiac grafts by SNMP has been investigated as an intervention to extend the short CIT of 4–6 h and overcome the irreversible myocardial damage in heart transplantation. Initial studies have shown that porcine hearts preserved statically at 21 °C with a novel heart-specific preservation solution (Somah) increased in ATP and creatine phosphate production, aerobic metabolism, heart function, and reduced edema when compared to standard preservation solution at 4 °C in an ex vivo reperfusion model [69,70]. This was further validated and tested for extending CIT in porcine heart transplantation following normothermic regional perfusion, which allows myocardial functional assessment and cardiac performance before procurement in situ [71]. Cardiac grafts preserved by SNMP showed improved myocardial function and a significant reduction in the levels of pro-inflammatory cytokines post-transplantation [71]. Furthermore, a recent study investigated the preservation of porcine neonatal hearts by SNMP due to the high scarcity and mortality in neonatal and pediatric heart transplantation. The cardiac grafts were perfused with the donor’s whole blood and modified Krebs–Henseleit solution with albumin for 10 h and subsequently orthotopically transplanted. The 10 h SNMP-treated grafts were functional and stable with no histological markers of damage post-transplantation [147]. These findings suggest that SNMP can be safely used to extend CIT in neonatal and adult hearts. However, this requires further investigation for validation.

## 7. Cellular and Molecular Changes in Organ Grafts During Subzero Preservation

Although graft preservation at 4 °C significantly slows down the metabolic rate of tissues, it does not halt metabolic activity entirely. At this temperature, cellular processes continue at a reduced pace, contributing to the gradual deterioration of graft quality over time. Every 10 °C drop in temperature leads to an approximate two-fold reduction in metabolic rate, highlighting the benefits of further cooling in organ preservation [148]. As a result, temperatures below 0 °C are currently being explored as a means to further suppress metabolic activity and extend viable storage times. However, a major limitation of subzero storage is the risk of tissue freezing associated with intracellular ice formation, which can cause severe structural damage. Preventing ice formation while maintaining low temperatures remains a central challenge in the development of effective subzero preservation techniques [149].

The main point of damage during subzero preservation is through mechanical damage induced by ice crystals and osmotic stress, leading to injury to the cell membrane integrity [150,151]. However, some studies are working on achieving a non-injurious partial-frozen state, as the location of ice formation is important in terms of tissue damage [152]. Ice formation in the extracellular space is thought to be less harmful than intracellular ice formation, since intracellular ice formation can lead to cell lysis. It is important to note that ice formation in the extracellular space draws water from the cellular compartment, further lowering the metabolic rate of cells. Interesting, various ectotherms exploit this situation to survive sub-zero temperatures. A well-studied example is *Rana sylvatica* (the wood frog), which produces a molar concentration of glucose to lower the freezing point in intracellular spaces, where 60% of the bodily fluids can remain frozen [153]. A notable strategy utilized by this species is that ice formation mostly occurs only within the blood vessels [154].

Another preservation technique currently under discussion involves lowering the temperature of tissues below 0 °C without ice formation. This is referred to as “supercooling”, which is usually achieved through the utilization of antifreeze reagents. These reagents are typically inspired by ectotherms, which synthesize proteins and other reagents to prevent ice formation in cold temperatures. Studies that investigate subzero storage utilize supercooling to circumvent tissue damage due to ice formation.

### 7.1. Renal Graft Preservation at Subzero Temperatures

An area of study involving subzero storage of donor kidneys is the identification of the main locations of ice formation in renal grafts, where kidneys from rabbits were utilized. Primary locations for ice formation were identified between the renal tubules in the extracellular space and within capillaries [151]. A study revealed the importance of the cooling rate of renal grafts in relation to successful subzero preservation. In this study, rabbit kidneys were first permeated with 2 M glycerol and cooled to −80 °C at cooling rates between 1 °C/h and 3 °C/min. At all cooling rates, ice was observed in the lumen of peritubular and glomerular capillaries and in the interstitial space, with preserved cellular ultrastructure [155]. In addition, there was no evidence of ice formation within the tubular lumen, the capsular spaces, or within the tubular cells [155]. However, all cooling rates resulted in microvasculature damage through intravascular ice formation. After rewarming the donor kidneys and removal of glycerol, the kidneys were auto-transplanted and were observed for 30 min. Interestingly, kidneys that were cooled at the slowest rate exhibited improved perfusion compared to those cooled at a more rapid rate [155]. In addition, rapidly cooled kidneys exhibited endothelial edema and hematuria, whereas slowly cooled kidneys exhibited only endothelial edema [155]. This study was notable, as it involved transplantation following subzero storage, accounting for the reperfusion injury after transplantation, although the long-term effects of the storage on the renal grafts were not assessed.

### 7.2. Non-Renal Graft Preservation at Subzero Temperatures

#### 7.2.1. Liver Graft Preservation at Subzero Temperatures

Several studies have investigated subzero preservation of liver grafts in preclinical and human discard models with the aim of extending CIT through freezing and supercooling approaches. Strategies utilized by freeze-tolerant organisms may not be plausible for mammalian tissues. One study specifically looked at the effects of freeze–thaw cycling on rat livers compared to livers preserved under the same thermal conditions and in the same solution in a supercooled state, without freezing using antifreeze proteins [156]. Findings from this study suggested that while hepatocytes survive high subzero cryopreservation, detachment of endothelial cells occurred upon thawing of the tissue, which was not observed in supercooled or standard-stored livers [156]. In comparison with *R. sylvatica*, where freezing within the vasculature was accomplished, it was also shown to have morphological differences [157]. For example, the radius of sinusoid capillaries in the *R. sylvatica* liver was five times greater than those in rat livers, also translating to a smaller surface area to volume ratio. A recent study involving partial freezing of rat liver grafts extended the storage period by 5 times. However, transplantation was only mimicked through an ex vivo model, and whether the grafts would be viable to support recipients in real transplantation was not assessed [158].

Various studies investigated supercooling of livers to extend their storage times through a variety of approaches. Some studies successfully used cryoprotective agents to prevent ice formation in rat livers preserved at −6 °C [159,160]. The authors used 3–O–methyl glucose (3–OMG) and polyethylene glycol (PEG) as cryoprotectants to prevent freezing in intracellular and extracellular spaces, respectively. However, a more clinically relevant study is required to push this preservation technique forward. A recent study demonstrated a protocol using discarded human livers, where freezing was prevented through minimization of air–liquid interfaces and use of antifreeze reagents to precondition the livers [149]. However, this protocol was only tested on livers that were repercussed ex vivo without viability assessment in a living system. While the results of these studies are clinically promising, a concern of utilizing antifreeze reagents is that they would need to be flushed out from the organ graft, and should not contact the recipient. A more recent study investigated supercooling of porcine livers through an isochoric (constant volume) system, including metastable supercooled solution without addition of antifreeze reagents, where liver grafts were preserved for up to 48 h at −2 °C [161]. This study also compared these supercooled livers to livers frozen at −2 °C for 48 h, where frozen liver grafts showed tissue disruption that was not observed in the supercooled grafts [161]. It is important to note that this study only assessed the livers based on morphological changes after the preservation period, and the authors did not perform any assessments on graft function or any changes following transplantation.

#### 7.2.2. Cardiac Graft Preservation at Subzero Temperatures

Subzero non-freezing models have been evaluated for cardiac grafts through various animal models. One study utilized a chamber that involved a variable magnetic field as an antifreeze system at −3 °C preserve rat hearts [162]. Cardiac grafts were preserved either at −3 °C with the use of the variable magnetic field, or at standard 4 °C temperature, followed by reperfusion for 2 h. Grafts that were supercooled had significantly improved coronary flow and higher function characterized by higher peak positive and peak negative dP/dT during reperfusion, and significantly less edema and higher ATP content after reperfusion compared to grafts that were preserved at 4 °C [162]. Using the magnetic field approach in porcine hearts, the same authors reported the same salutary effect, with improved mitochondrial integrity, higher ATP content, and less lactate accumulation at subzero temperature (−3 °C) compared to standard preservation temperature (4 °C) [163].

In a rat model of heterotopic heart transplantation, rat hearts were stored for 24 h either at −1.3 °C with antifreeze protein or at 4 °C with storage solution alone, followed by reperfusion for 60 min. Cardiac grafts that were supercooled had improved cardiomyocyte integrity compared to grafts preserved at 4 °C, as shown by electron microscopy [72]. The findings from all these experimental studies show that subzero storage represents a promising advancement in organ preservation, offering the potential to substantially extend CIT and improve graft viability by further reducing metabolic activity beyond what is achievable at the standard preservation temperature of 4 °C. However, the major barrier remains for the prevention of ice-induced injury, particularly from intracellular ice formation. Strategies such as supercooling and partial freezing, inspired by freeze-tolerant organisms such as *Rana sylvatica*, have demonstrated encouraging results in preclinical models, particularly for the kidney, heart, and liver. Supercooling approaches, especially those avoiding the use of exogenous antifreeze reagents, are especially attractive due to their clinical translatability. Despite notable improvements in structural preservation and short-term function across organ types, many studies still lack data on long-term graft function and viability post-transplantation. Therefore, future work should focus on translating these subzero preservation strategies into clinically viable protocols by incorporating functional transplantation studies, refining ice control methods, and ensuring biocompatibility of any additives used.

## 8. Conclusions

This review highlights the cellular and molecular changes induced by a range of preservation temperatures and techniques, and how these variables shape metabolic suppression, IRI, and transplant-specific outcomes across kidney and other solid organ grafts. However, despite an extensive and rapidly expanding the literature, no clear consensus emerges regarding an “optimal” storage temperature. The available evidence is heterogeneous across organs, preservation modalities, endpoints, and study designs, and it does not support a single temperature strategy that is consistently superior in all settings. Accordingly, while SCS at 4 °C remains the clinical standard due to its simplicity and cost-effectiveness, it also constrains wider utilization of marginal donor organs, limits extension of cold ischemia time CIT and restricts opportunities for graft viability assessment.

Accumulating preclinical and early clinical data suggest that alternatives—including HOPE (4 °C), moderate hypothermic preservation (10 °C), SNMP (20–32 °C), NMP (35–37 °C), and sub-zero storage—may confer advantages in selected contexts by preserving mitochondrial function, attenuating inflammatory injury, reducing the incidence of DGF and primary non-function, and enabling functional and viability assessments. Nevertheless, these approaches are not uniformly beneficial across all transplantable organs, donor types, or clinical scenarios, reinforcing the likelihood that temperature requirements are organ- and context-specific rather than universal.

Therefore, the field remains at a transitional stage: promising signals exist, but uncertainty persists, and broad clinical adoption of intermediate-temperature preservation will require larger, well-designed clinical trials to standardize protocols, confirm durability of long-term outcomes, reduce technical complexity, and address logistical and economic barriers. Future work should prioritize comparative, organ-specific studies that use harmonized outcome measures to clarify which temperatures and strategies best improve graft quality, safely extend CIT, increase graft utilization, and reduce post-transplant complications, ultimately improving long-term transplant outcomes.

## Figures and Tables

**Table 1:** Different storage methods and temperatures for various grafts, along with other models evaluated within the same studies cited in this article, are summarized in this Table.

Temperature Studied	Organ/Tissue	Method of Storage	Study Model	Conclusions/Effects
Hypothermic Preservation (4 °C) (Clinical Standard)	Kidney	HMP	Meta-analysis	Reduction in DGF compared to 4 °C SCS [40]
	Kidney	HOPE	Porcine	Improved early graft function compared to 4 °C SCS [41]
	Kidney	HOPE	Clinical trial	Less incidence of complications and graft failure compared to 4 °C HMP [42]
	Liver	HMP	Systematic review and meta-analysis	Less incidence of complications, improved one-year graft and patient survival compared to 4 °C SCS [43]
	Liver	HOPE	Systematic review and meta-analysis	Less incidence of complications compared to 4 °C SCS [44]
	Liver	HOPE	Clinical trial	Improved preservation of the tissue compared to 4 °C SCS [45]
	Heart	HMP	Meta-analysis	Reduction in primary graft dysfunction and lower 2-year mortality rate compared to 4 °C SCS [46]
	Heart	HMP	Human discards	Improved preservation, decreased inflammation compared to 4 °C SCS [47]
	Heart	HOPE	Clinical trial	Allows an increase in preservation time compared to 4 °C SCS [48]
	Lung	HMP	Canine	Improved function and reduced tissue edema compared to 4 °C SCS [49]
	Pancreas	HOPE	Clinical trial	Improved viability and ATP concentration compared to 4 °C SCS [50]
Hypothermic Preservation (10 °C)	Kidney	Static storage	Rat	Improved early graft function and organ viability, reduced injury and inflammation, and improved survival compared to 4 °C SCS [51]
	Kidney	Static storage	Rat	Less necrosis and apoptosis, reduced mitochondrial injury, and graft dysfunction compared to 4 °C SCS [52]
	Liver	Static oxygenated storage	Porcine	Improved vascular viability, function, and decreased DNA damage, improved metabolism compared to 4 °C SCS [53]
	Liver	HOPE	Rat	Improved ATP production and metabolism of anti-oxidative metabolites compared to 4 °C SCS [54]
	Liver	HOPE	Clinical trial	Improved preservation and improved early graft function compared to 4 °C SCS [45]
	Lung	Static storage	Porcine	Improved graft function, preservation of mitochondrial integrity, decreased cell death, decreased inflammatory response compared to 4 °C SCS [55]
	Lung	Static storage	Porcine	More production of cytoprotective metabolites, decreased cell death in the graft, and less apoptosis compared to lungs undergoing immediate transplant [56]
	Heart	Static storage	Clinical trial	Improved metabolism, allowing for longer storage periods compared to 4 °C SCS [57]
Normothermic Preservation (35–37 °C)	Kidney	Machine perfusion	Clinical trial	No change in rate of DGF between NMP and 4 °C SCS [58]
	Liver	Machine perfusion	Clinical trial	Decreased graft injury and early graft dysfunction compared to 4 °C SCS [59]
	Liver	Machine perfusion	Clinical trial	Decreased rate of early graft dysfunction compared to 4 °C SCS [60]
	Liver	Machine perfusion	Clinical trial	No significant difference compared to 4 °C SCS, though greater benefits in higher-risk donors [61]
	Lung	Machine perfusion	Clinical trial	Reduction in primary graft dysfunction, improved ATP levels, reduced inflammatory factors compared to 4 °C SCS [62]
	Heart	Machine perfusion	Clinical trial	Increases the donor pool for deceased donor cardiac grafts compared to 4 °C SCS [63]
	Heart	Machine perfusion	Clinical trial	No significant difference in rejection rate, cardiac allograft vasculopathy, and survival rate compared to 4 °C SCS [64]
Subnormothermic Preservation (20–32 °C)	Kidney	Machine perfusion	Porcine	Reduction in apoptosis and injury markers, improvement in renal flow and urine output with 22 °C storage compared to 15 °C and 37 °C [65]
	Kidney	Machine perfusion	Porcine	Improved renal graft function on post-operative day 7 for kidneys stored between 22 and 25 °C compared to 4 °C SCS [66]
	Liver	Machine perfusion	Human discards	Significantly lower levels of glutathione compared to NMP, reduction in antioxidative capacity [67]
	Lung	Machine perfusion	Rat	Improvement of graft function, oxygenation, ATP content, and reduction in inflammatory markers in lung grafts preserved at 21–28 °C compared to NMP [68]
	Heart	Static storage	Porcine	Storage at 21 °C with a novel preservation solution increased ATP and creatinine phosphate production, aerobic metabolism, graft function, and reduced edema compared to 4 °C SCS [69,70]
	Heart	Machine perfusion	Porcine	Improved myocardial preservation, improved diastolic function, decreased release of inflammatory markers through storage at 21 compared to 4 °C SCS [71]
Subzero Preservation	Heart	Static storage; supercooling	Rat	Improved cardiomyocyte integrity after reperfusion compared to 4 °C SCS [72]

## Data Availability

No new data were created or analyzed in this study. Data sharing is not applicable to this article.

## Abbreviations

**Abbreviation**	**Full Name**
IRI	Ischemia–reperfusion injury
SCS	Static cold storage
HMP	Hypothermic machine perfusion
HOPE	Hypothermic oxygenated machine perfusion
NMP	Normothermic machine perfusion
SNMP	Subnormothermic machine perfusion
CIT	Cold ischemic time
DGF	Delayed graft function
PNF	Primary non-function
MP	Machine perfusion
mPTP	Mitochondrial permeability transition pore
TLR	Toll-like receptor
PAMP	Pathogen-associated molecular pattern
ATP	Adenosine triphosphate
DNA	Deoxyribonucleic acid
mtDNA	Mitochondrial deoxyribonucleic acid
